# A199 BIOFILMS IN THE APPENDIX AND OTHER NON-INFLAMED SECTIONS OF THE COLON IN PEDIATRIC PATIENTS WITH INFLAMMATORY BOWEL DISEASES

**DOI:** 10.1093/jcag/gwac036.199

**Published:** 2023-03-07

**Authors:** N Arjomand Fard, M Bording-Jorgensen, J Webb, S Veniamin, C Cheng, T Perry, E Wine

**Affiliations:** 1 Physiology; 2 Pediatrics; 3 Medicine; 4 Surgery; 5 Pediatrics & Physiology, University of Alberta, Edmonton, Canada

## Abstract

**Background:**

Biofilms, aggregated bacteria colonizing the extracellular polymeric substances matrix, are associated with the mucosa of inflammatory bowel diseases (IBD) patients with some studies showing a mean density of the mucosal biofilms 2-fold higher in IBD patients than in controls. The appendix, which is a highly immune organ, seems to be involved in IBD pathogenesis.

**Purpose:**

In this study we aimed to evaluate biofilms in the appendix and other non-inflamed regions of the colon in pediatric IBD patients. We hypothesized that biofilms composed of pathobionts in these sections could drive inflammation in IBD patients.

**Method:**

Tissues from the appendix, peri-appendicular region, cecum, and ascending colon (ASC), collected from the resected colons of 9 pediatric IBD patients and one pediatric non-IBD patient, preserved in methanol Carnoy’s solution were processed and paraffin embedded. Combined fluorescence in situ hybridization (FISH) for biofilms (probe: EUB338) and immunofluorescence (IF) mucin staining (MUC2) identified biofilms and their location. Biofilm formation capacity of culturable bacteria (identified through 16S DNA Sanger sequencing) from these sections was measured. Interleukin (IL)-8 (pro-inflammatory chemokine) and IL-10 (anti-inflammatory cytokine) expressions were assessed by tissue qPCR.

**Result(s):**

FISH demonstrated biofilms in these sections, in close proximity to epithelial cells. We used a biofilm formation assay to assess the ability of the identified bacteria from these sections to form biofilms, illustrating their potential to colonize and evade host defense and potential antibiotics. We found that *Enterococcus avium* has a significantly higher ability to form biofilms than the negative control. IL-8 and IL-10 both had the highest expression level in the appendix and peri-appendicular region and the lowest in the ASC among all the patients, suggesting an active immune response. Mechanistic experiments are in progress to investigate the type of bacteria involved in the biofilms and their effects on the gut barrier integrity.

**Conclusion(s):**

Biofilms adjacent to the epithelium, especially in the appendix and peri-appendix, could interact with or invade epithelial cells. The elevated chemokine transcript level in the appendix could reflect the recruitment of immune cells to this section following bacterial invasion, resulting in the activation of the immune system. Identifying the bacteria involved in the biofilms and clarifying their characteristics will aid us in developing novel microbe-altering treatment strategies or personalized medicine.

**Disclosure of Interest:**

None Declared

